# Microtubule-associated protein tau is essential for long-term depression in the hippocampus

**DOI:** 10.1098/rstb.2013.0144

**Published:** 2014-01-05

**Authors:** Tetsuya Kimura, Daniel J. Whitcomb, Jihoon Jo, Philip Regan, Thomas Piers, Seonghoo Heo, Christopher Brown, Tsutomu Hashikawa, Miyuki Murayama, Heon Seok, Ioannis Sotiropoulos, Eunjoon Kim, Graham L. Collingridge, Akihiko Takashima, Kwangwook Cho

**Affiliations:** 1 Department of Aging Neurobiology, Center for Development of Advanced Medicine for Dementia, National Center for Geriatrics andGerontology, 35 Gengo, Morioka, Obu, Aichi 474-8522, Japan; 2 Henry Wellcome Laboratories for Integrative Neuroscience and Endocrinology, School of Clinical Sciences, Faculty of Medicine and Dentistry, University of Bristol, Whitson Street, Bristol BS1 3NY, UK; 3 Chonnam-Bristol Frontier Laboratory, Biomedical Research Institute, Chonnam National University Hospital, Gwangju 501-757, South Korea; 4 Centre for Synaptic Plasticity, University of Bristol, University Walk, Bristol BS8 1TD, UK; 5 School of Physiology and Pharmacology, University of Bristol, University Walk, Bristol BS8 1TD, UK; 6 Laboratory for Alzheimer's Disease, Brain Science Institute, RIKEN, 2-1 Hirosawa, Wako-shi, Saitama 351-0198, Japan; 7 Department of Biomedical Engineering, Jungwon University, 85 Munmu-ro, Goesan-gun, Chungcheongbuk-do 367-805, South Korea; 8 Life and Health Sciences Research Institute (ICVS), School of Health Sciences, Universidade do Minho, Campus de Gualtar, Braga 710-057, Portugal; 9 Center for Synaptic Brain Dysfunctions, Institute for Basic Science and Department of Biological Sciences, Korea Advanced Institute of Science and Technology, Daejeon 305-701, South Korea; 10 Department of Brain and Cognitive Sciences, College of Natural Sciences, Seoul National University, Gwanak-gu, Seoul 151-746, South Korea

**Keywords:** Alzheimer's disease, hippocampus, synaptic plasticity, long-term depression, tau

## Abstract

The microtubule-associated protein tau is a principal component of neurofibrillary tangles, and has been identified as a key molecule in Alzheimer's disease and other tauopathies. However, it is unknown how a protein that is primarily located in axons is involved in a disease that is believed to have a synaptic origin. To investigate a possible synaptic function of tau, we studied synaptic plasticity in the hippocampus and found a selective deficit in long-term depression (LTD) in tau knockout mice *in vivo* and *in vitro*, an effect that was replicated by RNAi knockdown of tau *in vitro*. We found that the induction of LTD is associated with the glycogen synthase kinase-3-mediated phosphorylation of tau. These observations demonstrate that tau has a critical physiological function in LTD.

## 1. Introduction

The microtubule-associated protein ‘tau’ (*MAPT*) gene is located on chromosome 17 and consists of 16 exons [1]. Alternative splicing leads to six isoforms of tau, all of which contain an amino-terminal projection domain and carboxy-terminal with microtubule-binding repeats [2]. Tau contains several critical serine and threonine residues, the phosphorylation of which regulates its binding affinity for microtubules [3,4]. It is believed that through this binding, tau has major roles in stabilizing microtubules [5]. During neuronal development, tau expression is increased in response to nerve growth factor [6], and subsequently enriched in axons, a process that is required for maintaining axon morphology [7]. The extent to which tau may have additional functions unrelated to axonal microtubule stabilization, however, is not known.

Tauopathies, such as Alzheimer's disease (AD), are characterized by widespread accumulation of hyperphosphorylated tau. Once hyperphosphorylated, tau is known to accumulate in somatodendritic compartments and forms the core component of neurofibrillary tangles (NFTs) [8]. It is generally believed that hyperphosphorylation of tau is the critical step in causing it to be missorted from the axon to dendrites, where it interferes with neuronal function [9]. Associated with this accumulation, there is a loss of synapses and eventually neurons [10,11]. However, the mechanism by which this occurs is unknown.

Increasing evidence suggests that in AD, synaptic dysfunction may initiate the cascades that result in cognitive impairment and neurodegeneration. For example, it is well established that oligomeric forms of β-amyloid (Aβ) induce a rapid alteration in synaptic plasticity, the process widely believed to underlie learning and memory in the brain [12]. More specifically, Aβ causes inhibition of long-term potentiation (LTP) and enhancement of long-term depression (LTD) in the hippocampus [13]. LTD involves the removal of AMPA receptors (AMPARs) from synapses leading to a reduction in synaptic efficiency, and can also result in the shrinkage and elimination of synapses [14]. Therefore, a shift in favour of LTD may lead to neurodegeneration. That such processes may be causally related to neurodegeneration in AD is suggested by the finding that key molecules that are associated with this disorder, such as glycogen synthase kinase (GSK)-3β and caspase-3, are required for the induction of LTD in the hippocampus [15–18] and mediate the Aβ inhibition of LTP [19]. Interestingly, recent evidence has shown that Aβ inhibition of LTP is absent in the tau knockout (KO) mouse [20]. These data, together with the observation that GSK-3β directly phosphorylates tau [15,18], suggest that tau may be a downstream effector of GSK-3β in LTD. Therefore, we decided to examine the role of tau in LTD in the hippocampus.

In this study, we found that in tau KO mice there is a loss of LTD, whereas LTP is not affected. Furthermore, knockdown of tau in hippocampal slices resulted in a complete loss of LTD in the absence of any direct discernible effects on synaptic transmission. We found that LTD was associated with the phosphorylation of tau by GSK-3β [18]. Collectively, these data suggest that tau phosphorylation is an essential component of LTD.

## 2. Results

### (a) Long-term depression is absent in *MAPT*^+/–^ and *MAPT*^–/–^ mice

**Figure 1.**
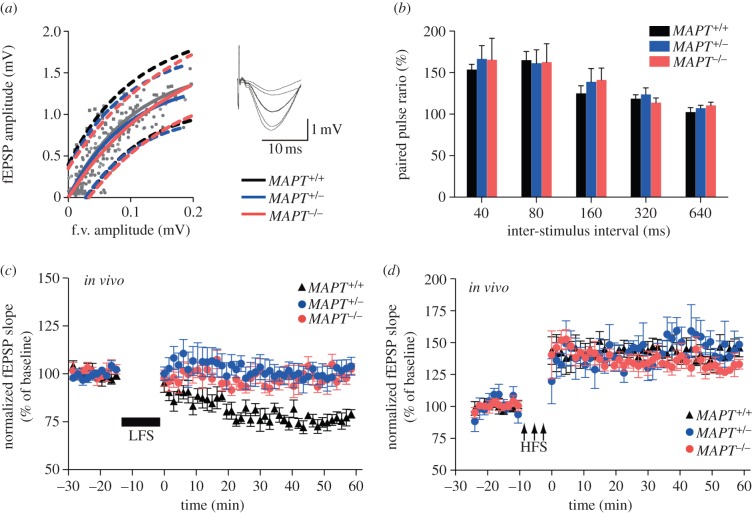
Tau is required for LTD *in vivo* in mice. (*a*) No differences are shown in synaptic transmission between *MAPT*^+/+^, *MAPT*^+/−^ and *MAPT*^−/−^ mice. The graph plots the fEPSP amplitude versus the fibre volley (f.v.) amplitude (stimulus intensity range: 10–100 μA, grey dots). Input-output curves show regression (continuous lines) and 95% confidence limits (dashed lines). (*b*) No differences are shown between *MAPT*^+/+^, *MAPT*^+/−^ and *MAPT*^−/−^ mice in paired-pulse facilitation at various inter-stimulus intervals. (*c*) LTD is absent in *MAPT*^+/−^ and *MAPT*^−/−^ mice. Pooled data from mice (age between 7 and 11 months; *MAPT*^+/+^: 76 ± 2%; *n* = 16; *MAPT*^+/−^: 96 ± 3%; *n* = 15; *MAPT*^−/−^: 98 ± 2%; *n* = 11; *p* < 0.001 in comparison with *MAPT*^+/+^ mice, Bonferroni's multiple comparison test). (*d*) No differences are shown in LTP between genotype. Pooled data from 7- to 11-month-old *MAPT*^+/+^ (141 ± 7% of baseline quantified at 60 min after the tetanus, *n* = 5), *MAPT*^+/−^ (141 ± 6%, *n* = 6) and *MAPT*^−/−^ mice (134 ± 3%, *n* = 4). HFS, high frequency stimulation. (Online version in colour.)

The physiological role of tau in the hippocampus was initially investigated using tau KO mice. We compared long-term synaptic plasticity in adult (7–11 months old) *MAPT*^+/+^, *MAPT*^+/–^ and *MAPT*^–/–^ mice. Because the tau kinase GSK-3β is required for LTD in the hippocampus [17], the primary focus of our investigation was on this form of synaptic plasticity. Field excitatory postsynaptic potentials (fEPSPs) were evoked in area CA1 of anaesthetized mice in response to electrical stimulation of the ipsilateral Schaffer collateral–commissural pathway. We found no differences in synaptic transmission between *MAPT*^+/+^, *MAPT*^+/–^ and *MAPT*^–/–^ mice, as assessed using input–output curves (figure 1*a*), and we observed no significant differences in paired-pulse facilitation over a range of inter-stimulus intervals (figure 1*b*). However, we found that while LTD could be readily induced in adult *MAPT*^+/+^ mice, it was completely absent in *MAPT*^+/–^ and *MAPT*^–/–^ mice (figure 1*c*). By contrast, similar levels of LTP were observed in the three genotypes (figure 1*d*). Therefore, tau is specifically required for LTD in the hippocampus *in vivo*.

**Figure 2.**
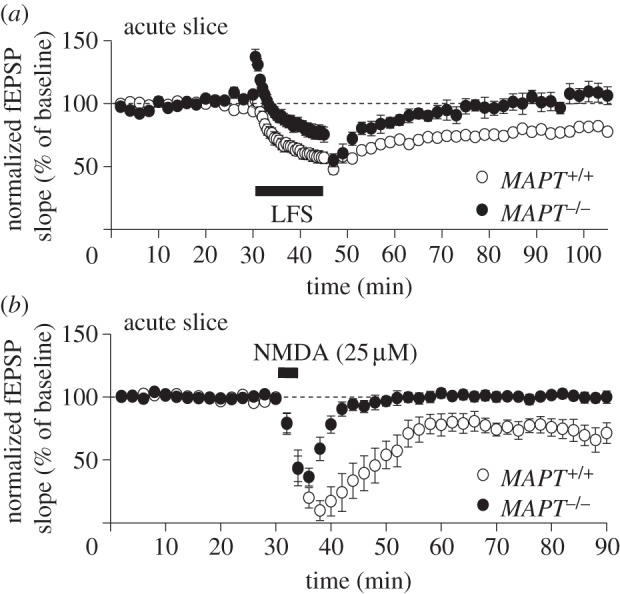
Tau is required for LTD *in vitro* in mice. (*a*) 1 Hz, 900 pulses induces LTD in *MAPT*^+/+^ mice (77 ± 3%, *n* = 6) but is absent in *MAPT*^−/−^ mice (106 ± 7%, *n* = 5). Pooled data from postnatal 14- to 17-day-old mice. (*b*) Bath application of NMDA (25 μM, 3 min) induces LTD in *MAPT*^+/+^ mice (71 ± 8%, *n* = 5) but no LTD in *MAPT*^−/−^ mice (99 ± 4%, *n* = 6). Pooled data from postnatal 14- to 17-day-old mice.

Next, we investigated LTD in acute brain slices from young (14- to 17-day-old) mice. Consistent with the observations *in vivo*, LTD was absent in slices prepared from *MAPT*^−/−^ mice but was readily induced in slices obtained from *MAPT*^+/+^ mice (figure 2*a*). We also investigated LTD induced by a brief application of NMDA (25 μM, 3 min) and found a specific deficit in slices from the *MAPT*^–/–^ mice (figure 2*b*). These results show that the LTD deficit in *MAPT*^–/–^ mice is apparent early in development and therefore is not directly associated with ageing.

### (b) Knockdown of tau by shRNA prevents long-term depression induction

**Figure 3.**
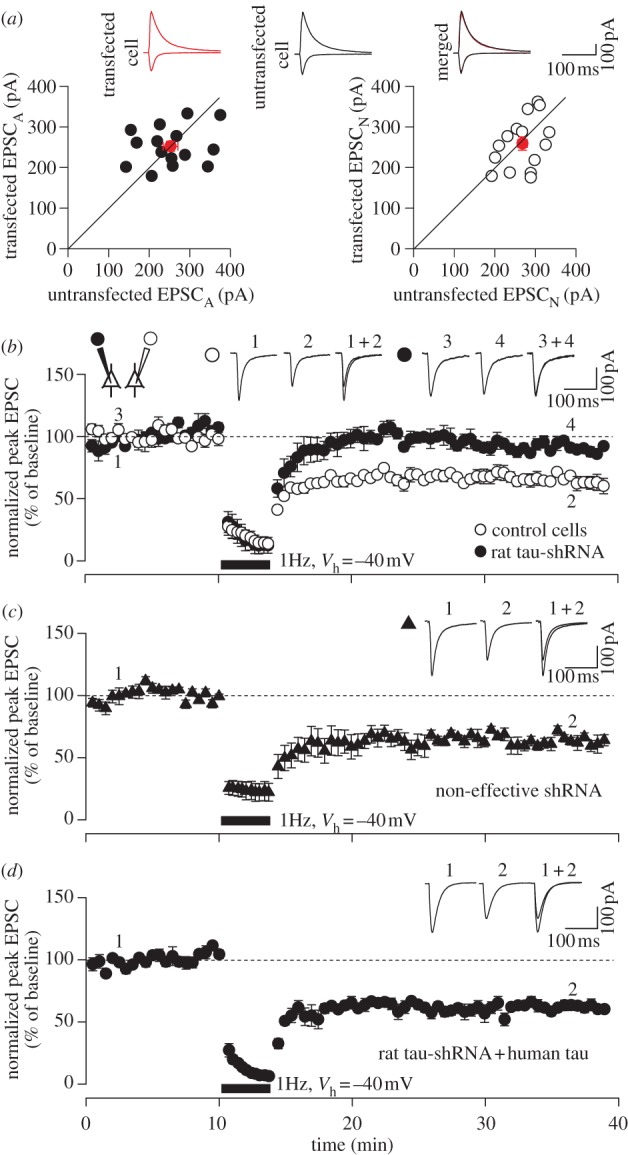
Knockdown of tau blocks LTD *in rats*. (*a*) Pairwise analysis of basal synaptic transmission between rat tau-shRNA expressing and untransfected neighbouring neurons, each obtained from independent slices. (*b*) Simultaneous dual-patch recordings were made from rat tau-shRNA-transfected and untransfected neighbouring cells. LFS was delivered (at time indicated by the bar) and LTD quantified 30 min later. (*c*) Data from scrambled tau-shRNA transfected cells. (*d*) Data from cells co-expressing rat tau-shRNA and human tau. Error bars indicate s.e.m. (Online version in colour.)

In these experiments, tau was absent or reduced throughout the life of the animals, potentially leading to developmental complications. Therefore, to investigate more directly whether tau is involved in the LTD process, we used an shRNA probe against rat tau and studied synaptic function in rat hippocampal organotypic slice cultures (figure 3). To study the effects of tau knockdown on synaptic transmission, simultaneous recordings of excitatory postsynaptic currents (EPSCs) were performed from tau-shRNA transfected and neighbouring untransfected neurons. There were no significant differences in AMPAR- and NMDA receptor (NMDAR)-mediated EPSCs (EPSC_A_ and EPSC_N_, respectively) between tau-shRNA transfected cells and neighbouring untransfected neurons (EPSC_A_ in transfected cells, 252 ± 12 pA; EPSC_A_ in untransfected cells, 253 ± 18 pA, *n* = 15 pairs, *p* > 0.05; EPSC_N_ in transfected cells, 256 ± 16 pA; EPSC_N_ in untransfected cells, 268 ± 12 pA, *n* = 15 pairs, *p* > 0.05; figure 3*a*). We next investigated whether tau-shRNA had any effect on LTD. Consistent with the LTD experiments in *MAPT*^+/–^ and *MAPT*^–/–^ mice, tau-shRNA blocked LTD, whereas LTD was routinely induced in simultaneously recorded, neighbouring untransfected cells (tau-shRNA transfected: 92 ± 3% of baseline; untransfected: 63 ± 7%, *n* = 5, *p* < 0.05, tau-shRNA versus control; figure 3*b*). The block of LTD was a specific consequence of the knockdown of endogenous tau, because expression of a non-effective, scrambled tau-shRNA had no effect on LTD (61 ± 7%, *n* = 5, *p* > 0.05, compared with control, figure 3*c*). Furthermore, the tau-shRNA-mediated LTD deficit was rescued by co-expression of human tau (60 ± 4%, *n* = 5, *p* > 0.05, compared with control, figure 3*d*), which was resistant to knockdown (rat tau-shRNA selectively reduces the expression of rat tau but has no effect on human tau; data not shown). These findings are consistent with our KO studies, confirming that tau is required for LTD induction. Taken together, our data show that tau is required for LTD across two species (rats and mice) and in both juvenile and adult tissue.

### (c) Tau is found in the postsynaptic compartment

**Figure 4.**
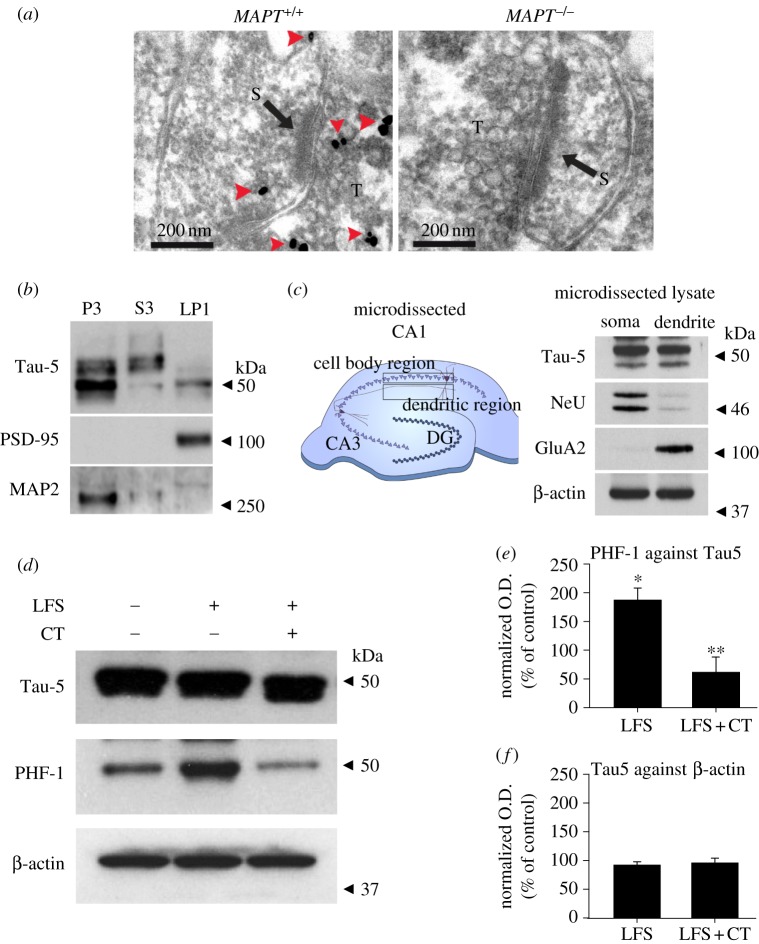
Tau is localized at the synapse and is phosphorylated during LTD. (*a*) Images of immunoelectron micrographs of hippocampal tissue obtained from *MAPT*^+/+^ (left panel; 4-month-old) and *MAPT*^−/−^ (right panel; 4-month-old) mice. Arrow shows synaptic density and arrowheads indicate tau. JM (rabbit polyclonal anti-tau antibody) and 10 nm gold particle conjugated secondary antibody gave positive signals in *MAPT*^+/+^ but not in *MAPT*^−/−^ mouse tissue. (*b*) Hippocampus of *MAPT*^+/+^ mouse (4 months old) was fractionated into a microsome/organelle fraction (P3), a cytoplasmic fraction (S3) and a PSD-95-rich fraction (LP1). MAP2 was mostly distributed in the P3 fraction. In comparison, tau (detected using Tau-5) was present in all fractions. (*c*) Schematic diagram of the microdissection procedure to separate the rat P24–28 CA1 somatic and dendritic regions. Western blotting shows strong expression of NeuN in the somatic region and of GluA2 in the dendritic region. Tau-5 blotting shows expression of tau in both the somatic and dendritic regions. (*d*) LFS causes an increase in phosphorylation of tau on Ser396/404 (PHF-1 epitope); this LFS-induced increase in phosphorylation of the PHF-1 epitope is attenuated by co-treatment with CT-99021 (CT; 1 μM). (*e*) Quantification of PHF-1 levels normalized to Tau-5 in the presence and absence of CT-99021 (control (CTR) versus LFS, *p < 0.05; LFS versus LFS + CT, **p < 0.01). (*f*) Quantification of Tau-5 expression, normalized to β-actin, in the presence and absence of CT-99021 (CTR versus LFS, *p* > 0.05; LFS versus LFS+CT, *p* > 0.05). Mann–Whitney non-parametric test was performed to identify changes in statistical significance. (Online version in colour.)

The finding that tau is required for LTD is surprising since LTD is generally considered to be mediated postsynaptically, via the synaptic removal of AMPARs, whereas tau is present primarily in axons. One possibility is that LTD causes the redistribution of tau to dendritic shafts and/or spines. An alternative possibility is that a small proportion of tau is normally expressed in a postsynaptic compartment and it is specifically this fraction that is involved in LTD. We explored the latter possibility in two ways. First, we used immunogold electron microscopy (EM) and compared the labelling of tissue from *MAPT*^+/+^ and *MAPT*^–/–^ mice (figure 4*a*). We could detect some immunoreactivity within dendritic spines of the *MAPT*^+/+^, but not *MAPT*^–/–^, mice. Second, we probed for the presence of tau, and another microtubule-associated protein (MAP2), in microsome/organelle (P3), cytoplasmic (S3) and synaptosomal (LP1) fractions prepared from the hippocampus of *MAPT*^+/+^ mice (figure 4*b*). As expected, tau and MAP2 were recovered in the P3 fraction and to a lesser extent in the S3 fraction. However, tau was additionally detected in the LP1 fraction. Thus, a proportion of tau is localized at a postsynaptic site where it could, in principle, function directly in LTD.

Because GSK-3β is a major tau kinase [15,18,21] and is activated during LTD [17], this seemed a likely candidate to mediate the physiological phosphorylation of tau. We hypothesized that the GSK-3β mediated phosphorylation of tau could be an important regulator of LTD. If this is indeed the case, then a prediction is that the induction of LTD should be associated with an increase in the phosphorylation of tau. To investigate this, we delivered low-frequency stimulation (LFS) and measured the phosphorylation status of tau in the CA1 microdissected dendritic region of rat hippocampal slices (figure 4*c*). We observed a dramatic increase in the phosphorylation of tau using PHF-1 (*p* < 0.01, *n* = 4, figure 4*d*,*e*), an antibody that recognizes phosphorylation at residues Ser396 and Ser404 [22], but observed no difference following LFS in the total levels of tau using Tau-5 (*p* > 0.05, *n* = 4, figure 4*d*,*f*), a phosphorylation-independent anti-tau antibody [23]. We next tested whether the phosphorylation of tau was due to activation of GSK-3 during LTD by applying a highly selective GSK-3β inhibitor, CT-99021, during LFS (figure 4*d*). This treatment eliminated the increase in tau phosphorylation (figure 4*d*,*e*), while having no effect on the total levels of tau (assessed using Tau-5; figure 4*d*,*f*). Collectively, these data demonstrate that LFS leads to the phosphorylation of tau in a GSK-3β-dependent manner, further supporting the idea that this phosphorylation event has a key role in the induction of LTD.

## 3. Discussion

In this study, we have provided several lines of complementary evidence to suggest that tau is important for LTD in the hippocampus. First, we have shown that LTD at CA1 synapses *in vivo* is not detectable in mice in which tau is absent or its expression levels are reduced. Second, we found that LTD was absent in slices acutely prepared from juvenile hippocampal tissues of *MAPT*^−/−^ mice. Third, we have demonstrated that knockdown of tau completely blocks LTD in organotypic-cultured slices. Fourth, we have shown that LFS used to elicit physiological LTD leads to enhanced phosphorylation of tau at the PHF-1 epitope, via a GSK-3β-dependent mechanism.

It is widely believed that tau, under normal conditions, is primarily involved in stabilizing microtubules in axons, and that the dysregulation of this function somehow leads to neuronal pathology [24]. The most prevalent form of such dysregulation occurs through the hyperphosphorylation of tau, which is involved in the generation of NFTs and plays a key role in neurodegenerative conditions such as AD [25]. Hyperphosphorylated tau is missorted to somatodendrites instead of axons, where it is known to accumulate [26–28]. Such missorting is assumed to contribute to neuronal pathology, because it positions tau where it can interfere directly with synaptic function. How tau becomes missorted is not known.

An alternative possibility is that some tau is normally present at synapses and it is this tau that is specifically associated with the neuropathology. Indeed, emerging evidence suggests that tau may be present in dendrites even in the absence of tauopathy [29] and that it could regulate interactions between scaffolding proteins and signalling pathways in the postsynaptic density (PSD) [30]. Furthermore, the localization of tau within the postsynaptic complex can be affected by NMDAR activation [31]. This raises an important question as to what the physiological function of tau in dendrites might be. Here, we have found, using both tau KO mice and RNAi in organotypic slices prepared from rats, that tau is required for LTD. This role is likely to be specific, because we found no evidence that tau is required for maintaining normal synaptic transmission or for LTP. Previous work [9,32] has shown that overexpression of tau may lead to inhibition of LTP. Based on the present findings, we propose that this may be because excess activation of tau induces a chronic form of LTD that is manifest as an impairment in LTP.

We also found that LFS, a physiological LTD induction protocol, resulted in the phosphorylation of tau and that this was dependent on GSK-3. Thus, tau is most probably a physiological substrate of GSK-3β during LTD. The next key question concerns the physiological downstream effectors of tau during LTD. At present, we can only speculate on this issue. Because tau is a microtuble-associated protein, and because microtubules may be involved in LTD [33], it is possible that tau is involved in the regulation of LTD-dependent microtubule dynamics.

Tau can be divided into a projection domain (towards the N-terminus, encompassing an acidic region and a proline-rich region) and a microtubule-binding domain (towards the C-terminus, including the microtubule-binding repeat region) [34], each having specific roles in the regulation of tau function [35,36]. Within these domains exist multiple regulatory sites of phosphorylation on serine/threonine residues. Our findings suggest a role in LTD for serine residues within the PHF epitope (Ser396/404). Consistent with our findings, Mondragon-Rodriguez *et al*. [31] recently reported a facilitation of Ser396/404 phosphorylation following NMDA treatment. Critically, Ser396/404 residues can both be phosphorylated by GSK-3β [37,38], an enzyme that is required for the induction of LTD [17]. However, it was unknown which, if any, of the potential GSK-3β phosphorylation sites on tau are phosphorylated during the physiological activation of this kinase. Our finding that phosphorylation of Ser396/404 following LFS is prevented by CT-99021 demonstrates that GSK-3β is upstream of tau in LTD and that this particular phosphorylation event probably has a physiological function.

## 4. Conclusion

We have shown that tau is required for NMDAR-dependent LTD is the hippocampus. Our data suggest a model whereby during LTD, activation of GSK-3β leads to phosphorylation of tau and this promotes LTD.

## 5. Methods

### (a) *In vivo* electrophysiology

Male C57/BL6J mice were used for all comparative KO experiments. *MAPT*^−/−^ and *MAPT*^+/−^ mice were maintained by backcrossing with C57/BL6J mice. Mice were individually housed and kept on a 12 h light/dark schedule. All mice had free access to food and water. Each mouse was anaesthetized with 3% isoflurane–air mixture, and fixed in a stereotaxic device (model 900, David Kopf Instruments, USA). After exposing the skull, a bipolar-stimulating electrode (two enamel-coated wires with 10 µm diameter and 200 kΩ impedance) was positioned into stratum radiatum of the left hippocampal CA1 area (−1.7 mm from bregma, 1.65 mm from medial, 1.3 mm depth) and a mono-polar recording electrode (0.5–1 MΩ) was placed 200 µm posterior to the stimulating electrode. The animal was maintained anaesthetized (1.5% isoflurane–air mixture) for at least 3 h (body temperature was kept at 36°C). For the fEPSP measurements, the electrical signal was amplified 100 times (ER-1, Cygnus Technology, USA), digitized (Digidata 1321A, Axon Instruments, Foster City, CA, USA) and processed on a computer. To induce LTP, 100 pulses at 100 Hz were applied three times (at 180 s intervals), and to induce LTD, 900 pulses at 1 Hz were delivered. The amplitude and slope of each recorded fEPSP was measured by a custom application based on MATLAB (version 8, Mathworks Inc., CA, USA). fEPSPs were analysed only when the maximal amplitude was over 1 mV, and the latency of the minimum peak from stimulus was shorter than 7 ms. Experiments were performed blindly.

### (b) *In vitro* electrophysiology

For *in vitro* electrophysiology experiments, acute hippocampal slices were obtained from P24 to P28 male Wistar rats or *MAPT*^−/−^ and *MAPT*^+/−^ mice. Animals were sacrificed by dislocation of the neck and then decapitated. The brain was rapidly removed and placed in ice-cold artificial cerebrospinal fluid (aCSF) containing (in mM): NaCl 124, KCl 3, NaHCO_3_ 26, NaH_2_PO_4_ 1.25, CaCl_2_ 2, MgSO_4_ 1 and d-glucose 10 (bubbled with 95% O_2_/5% CO_2_). Transverse hippocampal slices (400 µm thick) were prepared using a McIllwain tissue chopper (Mickle Laboratory Engineering Co. Ltd., Gomshall, UK). Hippocampal slices were stored in aCSF (20–25°C) for 1–2 h before transferring to the recording chamber, in which they were submerged in aCSF (30°C) flowing at 2 ml min^−1^. Extracellular field potentials were recorded in the CA1 region using glass electrodes containing NaCl (3 M). A stimulating electrode in CA2 was used to evoke field EPSPs (constant voltage, 100 µs duration, repeated at 30 s intervals). The slope of the evoked fEPSP was measured and expressed relative to the normalized preconditioning baseline. Data were captured and analysed using WinLTP (www.winltp.com). Experiments in which changes in the fibre volley occurred were discarded.

### (c) Hippocampal slice culture

Hippocampal slice cultures were prepared from 6- to 7-day-old male Wistar rats, as previously described [19]. Whole-cell patch clamp recordings of CA1 neurons transfected with shRNA plasmids were made 3–4 days following transfection. Using a biolistic Gene Gun (BioRad, USA), neurons were transfected with plasmids expressing shRNA against rat tau protein (OriGene Technologies, MD, USA). A mixture of four different tau (0N/3R, 0N/4R, 1N/4R and 2N/4R) shRNA constructs (1 : 1 : 1 : 1, in pGFP-V-RS vector) were used for tau silencing. A non-effective scrambled sequence shRNA was used as a negative control against tau-shRNA. EPSCs were recorded using a multi-clamp 700B amplifier (Axon Instruments). Recordings were carried out in a solution containing (in mM): NaCl 119, KCl 2.5, CaCl_2_ 4, MgCl_2_ 4, NaHCO_3_ 26, NaH_2_PO_4_ 1, glucose 11, picrotoxin 0.02 and 2-chloroadenosine 0.01, gassed with 5% CO_2_/95% O_2_, at pH 7.4. To induce LTD, 200 pulses at 1 Hz were delivered at a holding potential of −40 mV. AMPAR-mediated EPSC amplitude (EPSC_A_) was determined as the peak EPSC amplitude at a holding potential of −70 mV. NMDAR-mediated EPSC amplitude (EPSC_N_) was determined 50–70 ms after the EPSC_A_ peak at a holding potential of +40 mV. In some experiments, dual patch clamp recordings were made simultaneously from a pair of neighbouring CA1 pyramidal neurons, one transfected and the other untransfected. *n* values indicate number of cells, each obtained from independent slices. Error bars indicate s.e.m.

### (d) Tau constructs

0N3R and 2N4R human tau cDNAs were framed in pEGFP-C1 host vectors (Clontech, Mountain View, CA, USA), and provided by Drs R. Brandt (University of Ostanbrück, Germany) and S. Lovestone (King's College, UK).

### (e) Immunogold electron microscopy

Under deep pentobarbital anaesthesia, animals were perfused with 4% paraformaldehyde in 0.1 M cacodylate buffer (CB, pH 7.4). After further fixation of the brain at 4°C overnight, 300-μm-thick hippocampal slices were made. After incubation with blocking solution (5% normal goat serum in 0.1 M CB) for 1 h at room temperature, the slices were incubated with primary anti-tau antibody, JM (rabbit, 1 : 300), at 4°C for 2 days, followed by a secondary anti-rabbit IgG conjugated with FITC-gold (goat, Nanoprobes, NY, USA, 1 : 100) overnight. The slices were re-fixed with a mixture of 2.5% glutaraldehyde and 1% tannic acid at 4°C overnight. The gold signal enhancement procedure was performed according to the manufacturer's instruction (GoldEnhance-EM, Nanoprobes). After the osmication of slices (1% OsO_4_–1.5% potassium ferrocyanide in 0.1 M CB) at 4°C for 10 min, the slices were dehydrated, and embedded in epoxy resin. The stratum radiatum of CA1 region was examined electron microscopically (JEM-1200EX, JEOL, Japan) after metal-staining using uranium acetate and lead citrate.

### (f) Subcellular fractionation

Partial subcellular fractionation was performed on mouse hippocampus basically according to a previous report [39]. Postnuclear supernatant was subjected to centrifugation (12 500*g*), and divided into the crude synaptosomal fraction and synaptosome-depleted fraction. The crude synaptosomal fraction was further purified by hypotonic lysis and centrifugation (25 000*g*), and the resultant pellet was the PSD-95-rich synaptosome fraction (LP1). The synaptosome-depleted fraction was further subjected to ultracentrifugation (100 000*g*), and separated to the microsome fraction (P3) and cytoplasm fraction (S3). We used the following antibodies for experiments: NeuN, mouse monoclonal (Millipore; 1 : 1000) Tau-5, mouse monoclonal (Invitrogen; 1 : 500); PSD-95 (Millipore; 1 : 1000) and MAP2 (Millipore; 1 : 1000).

### (g) Low-frequency stimulation, microdissection and western blotting

Rat hippocampal slices from P24 to P28 were subjected to a standard 1 Hz, 900 pulses, LFS protocol (stimulation set at a predetermined intensity; 70% of the maximal fEPSP amplitude voltage stimulation), using a bipolar stimulation electrode, in the presence or absence of CT-99021 (1 μM). The dendritic CA1 region was then immediately dissected and snap frozen. Samples were lysed and SDS–PAGE was performed as previously shown [19]. PHF1 monoclonal antibody (kindly provided by Dr Peter Davies) was used at 1 : 1000 for western blot. Optical densities of immunoreactive bands were quantified using NIH ImageJ software (downloaded from http://rsb.info.nih.gov/ij/). *n* indicates the number of independent experiments from different animals.
